# Upstream ORF affects MYCN translation depending on exon 1b alternative splicing

**DOI:** 10.1186/1471-2407-9-445

**Published:** 2009-12-17

**Authors:** Roger Besançon, Sandrine Valsesia-Wittmann, Clara Locher, Céline Delloye-Bourgeois, Lydie Furhman, Giovani Tutrone, Christophe Bertrand, Anne-Catherine Jallas, Elisabeth Garin, Alain Puisieux

**Affiliations:** 1 INSERM UMR590, 28 rue Laënnec, Lyon, France; 2 Université de Lyon, université Lyon 1, Institut des Sciences Pharmaceutiques et Biologiques, 8 avenue Rockefeller, Lyon, 69008, France; 3 Centre Léon Bérard, Laboratoire de Recherche Translationnelle, 28 rue Laënnec, Lyon, France

## Abstract

**Background:**

The *MYCN *gene is transcribed into two major mRNAs: one full-length (*MYCN) *and one exon 1b-spliced (*MYCN*^Δ1*b*^) mRNA. But nothing is known about their respective ability to translate the MYCN protein.

**Methods:**

Plasmids were prepared to enable translation from the upstream (uORF) and major ORF of the two *MYCN *transcripts. Translation was studied after transfection in neuroblastoma SH-EP cell line. Impact of the upstream AUG on translation was evaluated after directed mutagenesis. Functional study with the two *MYCN *mRNAs was conducted by a cell viability assay. Existence of a new protein encoded by the *MYCN*^Δ1*b *^uORF was explored by designing a rabbit polyclonal antibody against a specific epitope of this protein.

**Results:**

Both are translated, but higher levels of protein were seen with *MYCN*^Δ1*b *^mRNA. An upstream ORF was shown to have positive cis-regulatory activity on translation from *MYCN *but not from *MYCN*^Δ1*b *^mRNA. In transfected SH-EP neuroblastoma cells, high MYCN dosage obtained with *MYCN*^Δ1*b *^mRNA translation induces an antiapoptotic effect after serum deprivation that was not observed with low MYCN expression obtained with *MYCN *mRNA. Here, we showed that MYCNOT: *MYCN *Overlap Transcript, a new protein of unknown function is translated from the upstream AUG of *MYCN*^Δ1*b *^mRNA.

**Conclusions:**

Existence of upstream ORF in *MYCN *transcripts leads to a new level of MYCN regulation. The resulting MYCN dosage has a weak but significant anti-apoptotic activity after intrinsic apoptosis induction.

## Background

**Figure 1:**
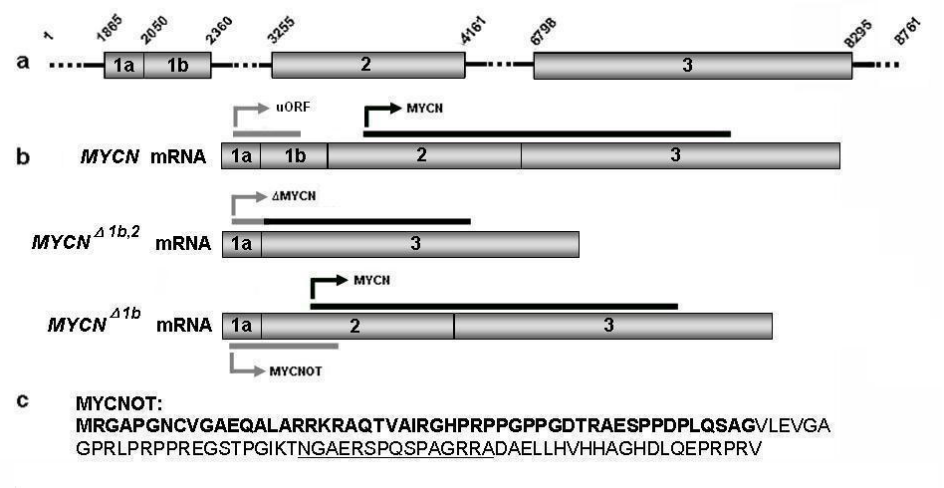
**The *MYCN *oncogene and encoded proteins**. **a) **Annotated sequence of *MYCN *according to Genbank Y00664. **b) **Alternative splicing and putative proteins encoded by *MYCN*. **c) **MYCNOT sequence. Bold aminoacids: shared homology with the putative ΔMYCN [4]. Underlined aminoacids: selected epitope for polyclonal antibody production in rabbit.

The *MYCN *gene, located in 2p23-24 [1] has been demonstrated to be composed of three exons (Fig. 1): exon 1a/b, exon 2 and exon 3, and the coding sequence for MYCN has been mapped to exons 2 and 3 [2]. Several transcripts have been described; one full length: *MYCN *mRNA and two alternatively spliced mRNAs; one containing exons 1a, 2 and 3: *MYCN*^Δ1*b *^mRNA [2,3] and the other containing exons 1a and 3: *MYCN*^Δ1*b*,2 ^mRNA [4]. Only *MYCN *and *MYCN*^Δ1*b *^transcripts might translate the MYCN protein. It was previously hypothesized but neither proven nor explored that an upstream AUG (uAUG) located in +1894 (according to GenBank: Y00664) of *MYCN*^Δ1*b *^mRNA might translate a protein (GenBank: AAG40001.1) [5]. Moreover, as proposed by van Bokhoven *et al*, *MYCN*^Δ1*b*,2 ^mRNA could be able to initiate translation of a new protein (ΔMYCN) from the same uAUG [4].

The existence of upstream open reading frames (uORFs) has been described in detail for many genes, and these uORFs could represent as much as half of the whole transcriptome [6,7]. Most studies have shown a negative cis-regulatory function of the major ORF [6-8].

MYCN, a basic helix-loop-helix transcription factor that belongs to the MYC family, was initially identified as a gene amplified in neuroblastoma, the most frequent paediatric extra-cranial solid tumour [9]. Numerous experiments have demonstrated its importance during ontogenesis. In the developing mouse embryo, MYCN is required for normal organogenesis of the heart, neural tube, spinal ganglia, visceral arches, liver, stomach, limb buds and eyes [10]. More precisely, MYCN is essential to maintain a population of undifferentiated and proliferating progenitor cells in the brain [11] and the distal lung epithelium [12] of mouse embryos. MYCN is also able, in chick embryos, to initiate the ventral migration of cells from the neural crest to the sympathetic ganglia and to induce their differentiation into neurons [13].

The overexpression of MYCN in the presence of deregulated H-ras has been shown to contribute to the neoplastic transformation of rat embryo cells, suggesting an oncogenic role of MYCN [14]. This oncogenic role was later confirmed using MYCN-transgenic mice: targeted expression of MYCN to neural crest-derived cells under the control of the *Tyrosine Hydroxylase *promoter leads to the development of neuroblastoma-like tumours in all homozygous mice [15].

The amplification and overexpression of MYCN primarily found in 25% of neuroblastomas are associated with advanced tumour stage, tumour progression and poor outcome [16]. *MYCN *can also be overexpressed in other tumours including medulloblastoma, retinoblastoma, small cell lung cancer, glioblastoma and other embryonal tumours [17] suggesting the implication of MYCN dosage in cancer development and aggressive expressivity. In single copy *MYCN *(SCN) neuroblastoma cell lines, MYCN has also been demonstrated to enhance proliferation after bFGF administration [18]. Contrariwise, the protein exhibits pro-apoptotic properties in particular conditions such as drug-triggered apoptosis [19,20] or induction of the death receptor machinery by TRAIL [21].

Thus, MYCN protein exhibits dual properties in proliferation and apoptosis, both in physiological and in pathological conditions.

In the present work, we evidenced a link between *MYCN *transcription and the level of MYCN translation. *In vitro *translation of the two coding mRNAs led to different levels of MYCN expression, with *MYCN*^Δ1*b *^mRNA being the most efficient. Their translation was differently regulated by an uORF located in the long 5' untranslated region of exon 1a/b. Moreover, we observed that the uORF of *MYCN *mRNA was able to up-regulate MYCN translation, whereas the uORF of *MYCN*^Δ1*b *^mRNA was not. Our results demonstrated a MYCN dosage effect in SH-EP cells in which high amounts of MYCN were anti-apoptotic after serum deprivation, compared to low levels or absence of MYCN. Finally, the uORF of *MYCN*^Δ1*b *^mRNA directed the translation of a new protein of unknown function, MYCNOT.

## Methods

### Cell culture

SH-EP cell line (an epithelial substrate-adherent Schwann-like clone issued from SK-N-SH neuroblastoma cell line[22] that do not express MYCN protein was cultured in RMPI 1640 (Sigma) supplemented with 10% foetal calf serum (GIBCO), penicillin G (200 IU/mL; GIBCO), streptomycin sulphate (200 μG/mL; GIBCO) and L-glutamine (2 mM; GIBCO) at 37°C in an atmosphere containing 5% CO_2_.

### Plasmids preparation

Plasmid preparations were based on the Invitrogen Gateway^® ^technology. Inserts were prepared from human foetal brain mRNAs (Clontech) with a forward primer specific to the very beginning of exon 1a/b at +1865 (according to Genbank Y00664) flanked with an attB1 sequence. Depending on the desired construct, three reverse primers extended with an attB2 sequence were designed. The first one, for *p-uORF/MYCN *was complimentary to the sequence of the penultimate nucleotides of the first stop codon of the *MYCN *mRNA in frame with the uAUG codon located at +1894. The second one, for *p-uORF/MYCN*^Δ1*b*^, was complimentary to the sequence of the penultimate nucleotides of the first stop codon of *MYCN*^Δ1*b *^mRNA in frame with the same uAUG codon. The third reverse primer, for *p-MYCN *and *p-MYCN*^Δ1*b*^, was complementary to the sequence of the penultimate nucleotides of the MYCN stop codon in exon 3. DNAs amplified from *MYCN *and *MYCN*^Δ1*b *^mRNAs were inserted into *pDEST40 *according to Invitrogen Gateway^® ^protocols with a C-terminal V5 epitope tag for visualizing protein translation.

The plasmids *p-MYCN*^*mut *^and *p-MYCN*^Δ1*b*, *mut *^were obtained respectively from *p-MYCN *and *p-MYCN*^Δ1*b *^by mutation of the upstream AUG located in +1894 into an AUC using the Quickchange^® ^Site-Directed Mutagenesis kit (Stratagene).

### Cell transfection

For translational studies, three independant experiments were conducted in duplicate: SH-EP cells were sowed at a density of 200000 cells in 6-wells plates and transfected the next day with 3.6 μg of purified *MYCN*-derived plasmids or control *pcDNA/GW-40/lacZ *(Invitrogen), 0.4 μg *p-EGFP *(Clontech) and 8 μL Lipofectamine 2000 (Invitrogen). Cells were harvested 24 hours after transfection.

For apoptosis experiments, three independent experiments were conducted in quadruplate: SH-EP cells were sowed at a density of 200000 cells in 6-wells plates and cultured in RMPI 1640 supplemented with 0.5% foetal calf serum, penicillin G (200 IU/mL), streptomycin sulphate (200 μG/mL) and L-glutamine (2 mM) for 48 hours at 37°C in an atmosphere containing 5% CO_2_, then transfected with 3.6 μg of *p-MYCN*, p-*MYCN*^Δ1*b *^or *pcDNA/GW-40/lacZ*, 0.4 μg *pEGFP *and 8 μL Lipofectamine 2000. After transfection, cells were cultured for an additional day in the same medium supplemented with 10% foetal calf serum.

### Western blotting

Proteins (40 μg) from cell lysates or from a human foetal and adult brain (protein Medley™; BD Biosciences) were separated by SDS-PAGE and transferred to 0.2 μm-nitrocellulose membranes (Biorad). Protein levels were determined using:

- a mouse anti-V5 epitope monoclonal antibody (1:5000, Invitrogen),

- a mouse anti-human β-ACTIN monoclonal antibody (1:200000, Sigma),

- a rabbit anti-NGAERSPQSPAGRRA [anti-MYCNOT] peptide polyclonal antibody (1:100, Eurogentec), whose specificity was checked by manufacturer

- a mouse anti-human MYCN monoclonal antibody, clone B8.4.B (1:1000, BD Pharmingen).

Primary antibodies were detected using goat anti-mouse (1:10000, Sigma) or anti-rabbit (1:3000, Sigma) horseradish peroxidase-conjugated secondary antibodies, and visualized using an enhanced chemiluminescence HRP substrate (Millipore).

### Cell viability assay

Cell viability was measured with Uptiblue viable cell counting reagent (Uptima) before serum deprivation and one day after transfection: cells were incubated for two hours after addition of 10% Uptiblue, fluorescence was measured with 530 nm excitation wavelength and 590 nm emission wavelength.

A one-sided student-t test was performed on the serum deprivation-induced apoptosis calculated as the fluorescence difference between the two measures.

## Results

### *MYCN *and *MYCN*^Δ1*b *^mRNAs are differently translated

**Figure 2:**
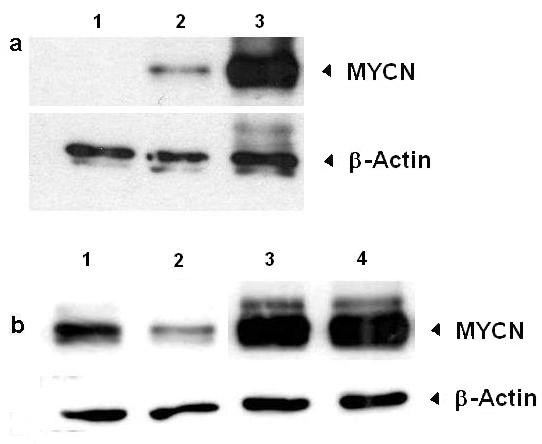
**Exon 1b-splicing, uAUG and MYCN translation**. **a) **Western blot analysis of 40 μg SH-EP neuroblastoma cells transfected with either control *pcDNA/GW40/lacZ *(lane 1), *p-MYCN *(lane 2) or *p-MYCN*^Δ1*b *^(lane 3). Results obtained with -upper panel: a monoclonal antibody against MYCN (1:1000) and -lower panel: a monoclonal antibody against β-Actin (1:10000). **b) **Western blot analysis of 40 μg SH-EP cells transfected with either *p-MYCN *(lane 1), *p-MYCN*^*mut *^(lane 2), *p-MYCN*^Δ1*b *^(lane 3) or *p-MYCN*^Δ1*b*, *mut *^(lane 4). Results obtained with -upper panel: the anti-MYCN antibody (1:1000) and -lower panel: the anti β-Actin antibody (1:10000).

Since alternative splicing of exon 1b affects a potential upstream open reading frame (uORF), we speculated that it could influence the translation of MYCN protein. Vectors *p-MYCN *and *p-MYCN*^Δ1*b *^were prepared to allow the translation of MYCN respectively from these two mRNAs. In SH-EP neuroblastoma cells, transfection assays followed by Western blot showed no MYCN expression in presence of the control plasmid (*pcDNA/GW40/lacZ*), weak but detectable translation from *p-MYCN*, whereas a very high amount of protein was obtained with *p-MYCN*^Δ1*b *^(Fig. 2a). The observed differences are not a simple consequence of experimental variations in either transfection efficacy, because cotransfection of *p-EGFP *induced no difference in the number of GFP-expressing cells between experiments (data not shown), or RNA transcription or stability, because semi quantitative RT-PCR analysis showed no difference in the levels of *MYCN *and *MYCN*^Δ1*b *^transcripts brought by plasmids (data not shown). Upstream ORF is usually known for *cis*-acting inhibitory effect on translation of the major protein. To test this hypothesis, the upstream AUG was mutated to AUC to produce *p-MYCN*^*mut *^and *p-MYCN*^Δ1*b*, *mut *^vectors. MYCN levels in SH-EP cells clearly diminished when it was translated from *MYCN *transcripts, but not from *MYCN*^Δ1*b *^transcripts (Fig. 2b).

### High MYCN dosage enhances cell viability

To explore the effect of MYCN dosage, SH-EP cells were subjected to apoptosis by 48-hour serum deprivation, then transfected with either control *pcDNA/GW40/lacZ*, *p-MYCN*, or *p-MYCN*^Δ1*b *^plasmids. As shown in Table 1, when SH-EP cells did not express (*pcDNA/GW-40/lacZ *transfected cells) or expressed low amount (*p-MYCN *transfected cells) of MYCN protein, serum deprivation induced similar cell loss (respectively 35.56% +/- 6.68% and 34.94 +/- 7.19%; p = 0.60). When SH-EP cells expressed high levels (*p-MYCN*^Δ1*b *^transfected cells) of MYCN protein, apoptosis (26.72% +/- 8.09%) was significantly (p = 0.01 and p = 0.02 respectively compared to previous conditions) reduced by one third.

**Table 1:** MYCN dose-dependant anti-apoptotic effect after serum deprivation.

Transfected plasmids	Cell viability (%)		
*pcDNA/GW-40/lacZ*	64.44 +/- 6.68

*pMYCN*	65.06 +/- 7.19^a^

*pMYCN*^Δ1*b*^	73.27 +/- 8.09^b,c^

MYCN dose-dependant enhanced SH-EP viability after serum deprivation measured with Uptiblue viable cell counting reagent (Uptima). ^a^ p= 0.60 compared with* pcDNA/GW-40/lacZ* ; ^b ^p= 0.01 compared with *pcDNA/GW-40/lacZ* ;^ c^ p= 0.02 compared with *pMYCN*

### MYCNOT is translated from a upstream AUG of *MYCN*^Δ1*b *^mRNA

**Figure 3:**
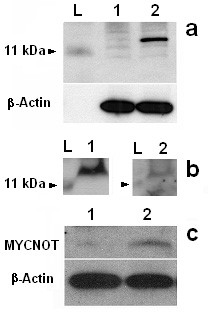
**Translation of uORF and existence of MYCNOT**. **a) **SH-EP cells were transfected with either *p-uORF/MYCN *(lane 1) or *p-uORF/MYCN*^Δ1*b *^(lane 2). Western blot was conducted on 40 μg proteins, 48 hours after transfection with -upper panel: a monoclonal antibody against the V5 epitope (1:300) and -lower panel: the anti β-Actin antibody (1:10000). L: protein ladder; **b) **SH-EP cells were transfected with *p-uORF/MYCN*^Δ1*b*^. Western blot was conducted on 40 μg protein, 48 hours after transfection with -left panel: a monoclonal antibody against the V5 epitope (1:300) and -right panel: a polyclonal rabbit anti-NGAERSPQSPAGRRA peptide [anti-MYCNOT] polyclonal antibody (1:100) L: protein ladder; **c) **Western blot analysis of 40 μg protein extracts from adult (lane 1) and foetal (lane 2) brain. Results obtained with -upper panel: the anti-MYCNOT antibody (1:100) and -lower panel: the anti β-Actin antibody (1:10000).

We speculated that uAUG, located in +1894 (according to GenBank: Y00664, may initiate the translation and we assessed the importance of alternative splicing of exon 1b. In SH-EP cells, the transfection of *p*-*uORF/MYCN*^Δ1*b*^, which contains the uORF of *MYCN*^Δ*1b *^mRNA, allowed the production of a V5 epitope-tagged protein of approximately 15 kDa, whereas the transfection of *p-uORF/Myc*, which contains the uORF of the full length *MYCN *transcript, did not (Fig. 3a). This 11.8 kDa new protein was named MYCNOT: *MYCN *Overlap Transcript.

To validate the existence of endogenous MYCNOT, we performed a Western blot analysis using a specifically designed polyclonal antibody raised in rabbit against a N-terminal epitope (NGAERSPQSPAGRRA) [anti-MYCNOT] of the hypothetical protein. The designed MYCNOT antibody was able to detect in transfected SH-EP cells with *p*-*uORF/MYCN*^Δ1*b *^the same protein detected with the V5 epitope antibody (Fig. 3b), The anti-MYCNOT antibody was able to detect a ~12 kDa protein in foetal but not in adult brain (Fig. 3c), thus confirming the existence of an endogenous MYCNOT protein. An analysis of MYCNOT sequence with BLAST and PROSITE software did not show any sequence or motif homology.

## Discussion

Because upstream ORF are known to influence protein translation [23], we investigated the MYCN translation from the full length *MYCN *mRNA and the exon 1b-spliced *MYCN*^Δ1*b *^mRNA. Only these two mRNAs are able to translate the MYCN protein. More recently, in 2005, van Bokhoven *et al *described a new exon 1a/2-spliced mRNA able to translate a new protein named ΔMYCN [4]. In SH-EP cells, we demonstrated that both *MYCN *and *MYCN*^Δ1*b *^were able to translate the MYCN protein, but MYCN translation from *MYCN*^Δ1*b *^mRNA was much more efficient. This difference may not be attributed to a differential stability of the two transcripts because they have the same half-life of approximatively 15 minutes [2]. But, that could be due to differences in the regulating activities of uORFs in the two mRNAs. Because, the coding AUG is several hundreds nucleotides after the stop codon of the uORF in *MYCN *mRNA compared to the overlapping uORF in *MYCN*^Δ1*b *^transcript, we should have expected a higher translation of MYCN protein from full length mRNA due to reinitiation [6,23], but as already demonstrated, this mRNA contains an Internal Ribosome Entry Site (IRES) that could explain the higher initiation of translation at the major AUG in *MYCN*^Δ1*b *^mRNA [5,24,25].

Upstream ORFs are usually known for their *cis*-acting inhibitory activity on the translation of the major protein [7,26-30]. To test this hypothesis, uAUG was mutated into AUC. This mutation had no effect on MYCN translation from *MYCN*^Δ1*b *^mRNA but significantly impacted the translation from *MYCN *mRNA. Thus, *MYCN *uORFs have different cis-regulatory activities in MYCN translation depending on the alternative splicing of exon 1b. In our conditions, the uORF of the *MYCN*^Δ1*b *^mRNA did not affect MYCN translation. Alternatively, the uORF transcribed from full length *MYCN *mRNA has cis-enhancing activity on MYCN translation. To our knowledge, only one team has shown that an uORF is able to augment the translation of the HIV-1 Env protein from the HIV-1 mRNA [30]. Taken together, our results suggest the existence of a new level of MYCN regulation. One hypothesis should be further explored: in cap-dependent conditions, low-level MYCN would be produced from the full length mRNA and this translation would be sustained by the activity of the uORF whereas in IRES-dependent conditions, high-level MYCN would be produced from exon 1b-spliced mRNA, independently of the uORF.

Numerous experiments have demonstrated important ontogenic [31] and oncogenic [14] roles. The amplification and overexpression of *MYCN *primarily found in 25% of neuroblastomas [16] and in other tumours including medulloblastoma, retinoblastoma, small cell lung cancer, glioblastoma and other embryonal tumours [17] suggest the implication of MYCN dosage in cancer development and aggressive expressivity.

Our results showed that, in transfected SH-EP neuroblastoma cells, high MYCN dosage obtained with *MYCN*^Δ1*b *^mRNA translation induces a weak but significant antiapoptotic effect after serum deprivation that was not observed with low MYCN expression obtained with *MYCN *mRNA. Our results do not fit the accepted pro-apoptotic properties of MYCN: using a MYCN-inducible SH-EP Tet21/N cell line, it has been demonstrated that MYCN overexpression enhances either doxorubicin- or platinum-induced apoptosis [19]. Using a similar approach, Cui et al have shown that the transfection of SH-EP cells with a *pBabe-hygro/MYCN *vector sensitises the cells to TRAIL-triggered apoptosis[21]. But, as we did, Jasty *et al *have reported a significant increase of cell viability with high MYCN expression after 6 days of serum deprivation [32]. These different results and ours suggest that MYCN overexpression in SH-EP cells might have different effects depending on the existence of an intrinsic (serum deprivation) or extrinsic (TRAIL or drug-induced apoptosis) pathway-mediated apoptosis. Thus, MYCN dosage, depending on translation of the alternatively exon 1b splicing mRNA, enhances cell viability after intrinsic pathway-mediated apoptosis.

As previously proposed, the uAUG codon located in exon 1a may initiate the translation of ΔMYCN, a new isoform of the MYCN protein [4]. It has also been hypothesized, but not proven at this time, that this uAUG is able to translate a protein (GenBank: AAG40001.1) [5]. We now demonstrated the existence of this 11.8 kDa protein and named it MYCNOT: *MYCN *Overlap Transcript. Even if uORFs appear to apply to as much as 50% of the transcriptome [6,7,23], only few papers have reported their translation in *in-vitro *experiments using chimeric tag-fusion proteins [23]. This is particularly true for the *MYC *gene, to which *MYCN *is related, which presents several uORFs: one uORF in exon 1 conducts to the translation of a protein of unknown function called MYCHEX1 [33]. To our knowledge, only one team, using two-dimensional chromatography and mass spectrometry analysis, has been able to prove the existence of eight proteins encoded by uORFs in human K562 and HEK293 cell lines [34]. Moreover, these small proteins (< 20 kDa) were not subject to rapid proteasome degradation suggesting functional activity [34]. The MYCNOT protein, which has no homology with MYCN, other known proteins or protein motifs, shares exon 1a-encoded amino acids with the putative ΔMYCN previously described [4]. Because MYCNOT is translated in foetal but not adult brain, we may hypothesize that it plays a role during neural development. Our results need further analysis to determine the functions of MYCNOT and might provide new insights into MYCN regulation.

## Conclusion

The *MYCN *gene is transcribed into two major mRNAs which are differently translated. Higher levels of protein were seen with *MYCN*^Δ1*b *^mRNA. Existence of an upstream ORF was shown to have positive cis-regulatory activity on translation from *MYCN *but not from *MYCN*^Δ1*b *^mRNA. The high MYCN dosage obtained with *MYCN*^Δ1*b *^mRNA translation induces, in transfected SH-EP neuroblastoma cells, a weak but significant antiapoptotic effect after serum deprivation that was not observed with low MYCN expression.

Here, we show the translation of a new protein of unknown function from the upstream AUG of *MYCN*^Δ1*b *^mRNA.

In conclusion, further studies are now needed to explore the exact relationship between alternative splicing of exon 1b, MYCN dosage, and the mechanism, either extrinsic or intrinsic, underlying apoptosis.

## Competing interests

The authors declare that they have no competing interests.

## Authors' contributions

RB carried out the design of the study and manuscript writing and participated in plasmids preparation, cell culture and Western blotting experiments. SWV participated to design of the study and manuscript writing. CL participated to cell culture and Western blotting experiments. CDB participated to plasmids preparation, cell culture and Western blotting experiments. GT participated to cell culture and Western blotting experiments. ACJ participated to cell culture and Western blotting experiments. EG participated to cell culture and Western blotting experiments. AP participated to design of the study and manuscript writing. All authors read and approved the final manuscript."

## Pre-publication history

The pre-publication history for this paper can be accessed here:

http://www.biomedcentral.com/1471-2407/9/445/prepub
